# Supplementary material for: Ageing impacts extracellular matrix turnover and remodelling in the kidney
Source: Matrix Biol Plus. 2026 May 15;30:100197. doi: 10.1016/j.mbplus.2026.100197 (PMC13213686; doi:10.1016/j.mbplus.2026.100197)
Supplement: Supplementary Data 1 — Supplementary Figure 1: Principal Components Analysis of normalised abundance and heavy-to-light ratios, distribution of total kidney matrix abundance, and volcano plots showing fold-changes in matrix protein abundance in 8-, 52-, and 78-week mice compared to 22-week mice in the kidney matrix-enriched fraction. Supplementary Figure 2: Volcano plots showing fold-changes in half-lives of kidney matrix proteins in the matrix-enriched fraction from 8-, 52-, and 78-week mice compared to 22-week mice. Supplementary Figure 3: Venn diagram showing kidney matrix proteins with significantly structure-associated differences identified by peptide location fingerprinting analysis in 8-, 52-, and 78-week mice relative to 22-week mice. [file mmc1.docx]

**Ageing impacts extracellular matrix turnover and remodelling in the kidney**

***Authors***

Rebecca Preston^1,2^, Anna Hoyle^3^, Alana Stevenson Harris^1,2^, Emily Williams^1,2^, Tess Birtles^4^, Joan Chang^2,5^, Joe Swift^1,2^, Alexander Eckersley^2,4^, Rachel Lennon^1,2,6^

***Affiliations***

1. *Division of Cell Matrix Biology & Regenerative Medicine, School of Biological Sciences, Faculty of Biology Medicine and Health, The University of Manchester, Manchester Academic Health Science Centre, Manchester, M13 9PT, UK.*
2. *Manchester Cell-Matrix Centre, School of Biological Sciences, Faculty of Biology Medicine and Health, The University of Manchester, Manchester Academic Health Science Centre, Manchester, M13 9PT, UK.*
3. *Kennedy Institute of Rheumatology, The University of Oxford, Oxford, OX3 7FY, UK*
4. *Division of Musculoskeletal & Dermatological Sciences, School of Biological Sciences, Faculty of Biology Medicine and Health, The University of Manchester, Manchester Academic Health Science Centre, Manchester, M13 9PT, UK.*
5. *Division of Molecular & Cellular Function, School of Biological Sciences, Faculty of Biology Medicine and Health, The University of Manchester, Manchester Academic Health Science Centre, Manchester, M13 9PT, UK.*
6. *Department of Paediatric Nephrology, Royal Manchester Children’s Hospital, Manchester University Hospitals NHS Foundation Trust, Manchester Academic Health Science Centre, Manchester, M13 9WL, UK.*

***Corresponding author email address***

Rachel.lennon@manchester.ac.uk

**
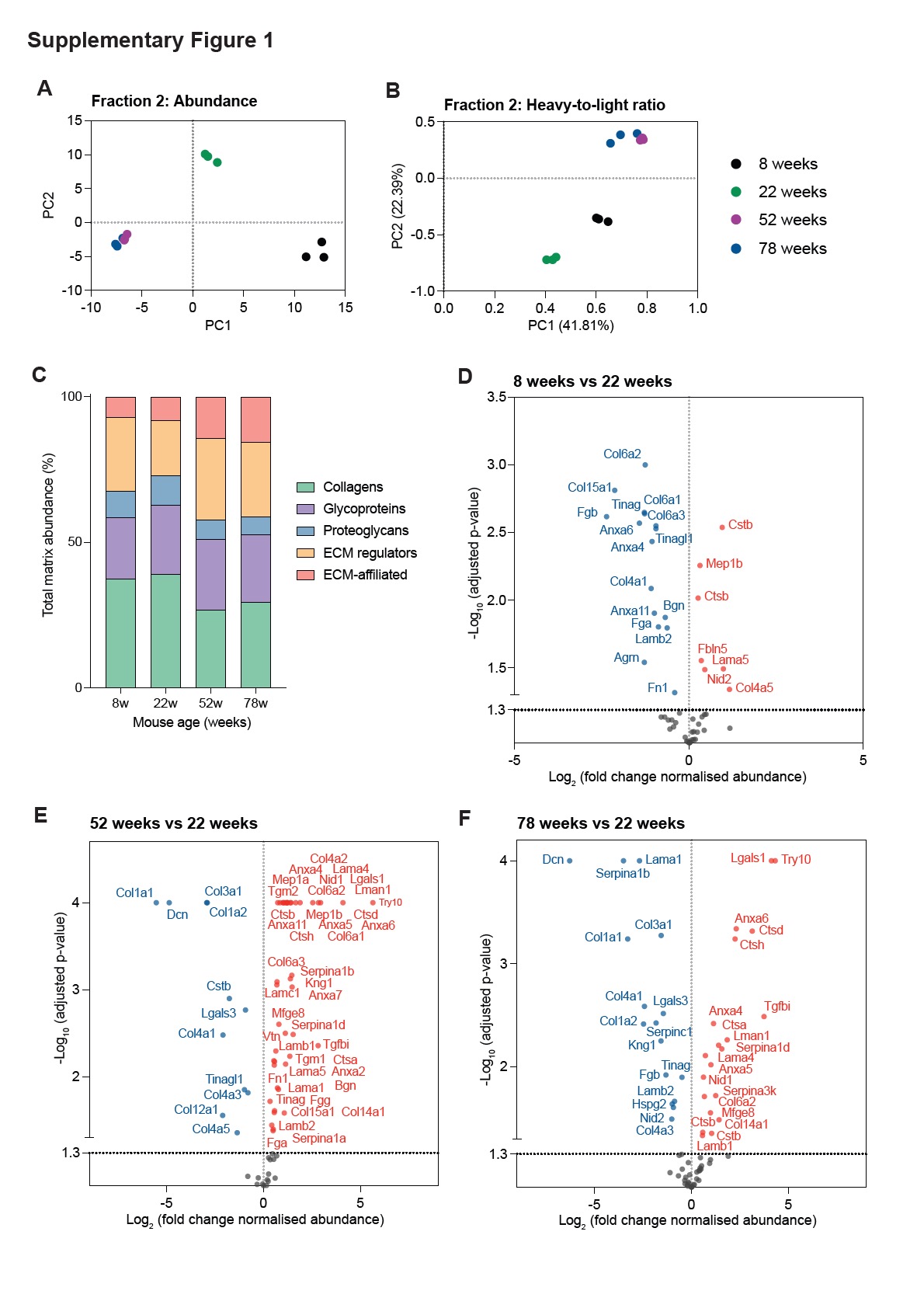
**

**Supplementary Figure 1:** Principal components analysis (PCA) of **A**) normalised abundance, and **B**) heavy-to-light ratios from the kidney matrix-enriched fraction from 8-, 22-, 52-, and 78-week mice. **C**) Distribution of total kidney matrix abundance derived from heavy and light MS1 ion intensities from matrisome proteins in the matrix-enriched fraction from 8-, 22-, 52- and 78-week-old mice demonstrating a relative reduction in core matrix proteins with age. **D**) Volcano plot showing fold-changes in abundance of kidney matrix proteins identified in the matrix-enriched fraction from 8- week compared to 22-week mice. **E**) Volcano plot showing fold-changes in abundance of kidney matrix proteins identified in the matrix-enriched fraction from 52- week compared to 22-week mice. **F**) Volcano plot showing fold-changes in abundance of kidney matrix proteins identified in the matrix-enriched fraction from 78- week compared to 22-week mice. For (D), (E), and (F), matrix proteins with significantly increased abundance in 8-, 52-, or 78-week mice are shown in red and matrix proteins with significantly decreased abundance in 8-, 52-, or 78-week mice are shown in blue. Dotted black line represents significance threshold (adjusted p<0.05). N=3 biological replicates per condition for all analysis.

**
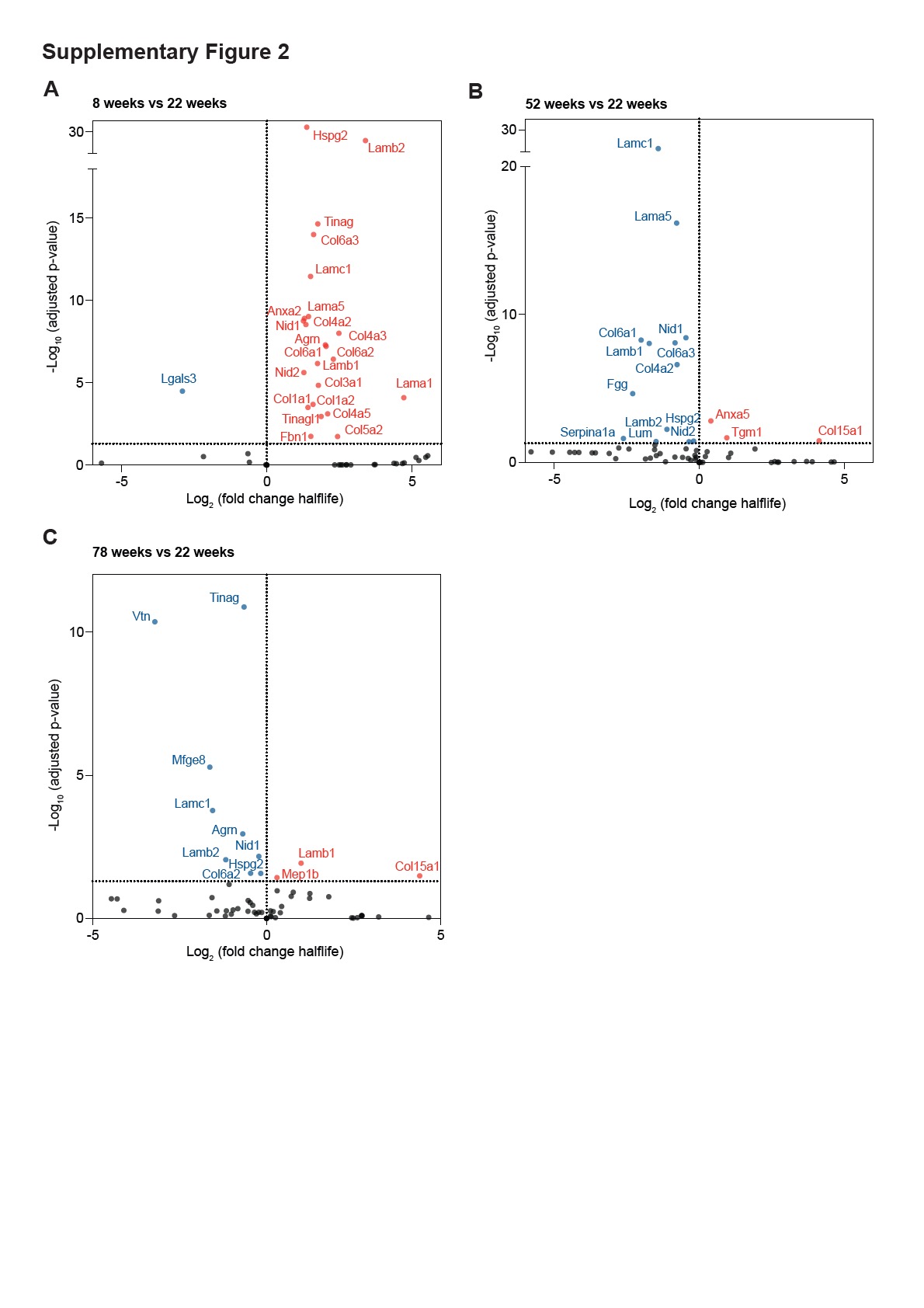
**

**Supplementary Figure 2:** Volcano plot showing fold changes in half-lives of kidney matrix proteins identified in the matrix-enriched fraction from **A**) 8-week vs 22-week, **B**) 52-week vs 22-week, and **C**) 78-week vs 22-week mice. Matrix proteins with significantly shorter half-lives (faster turnover) in 8-, 52-, or 78-week mice are shown in red and matrix proteins with significantly longer half-lives (slower turnover) in 8-, 52-, or 78-week mice are shown in blue. Dotted black line represents significance threshold (adjusted p<0.05). N=3 biological replicates per condition for all analysis.

**
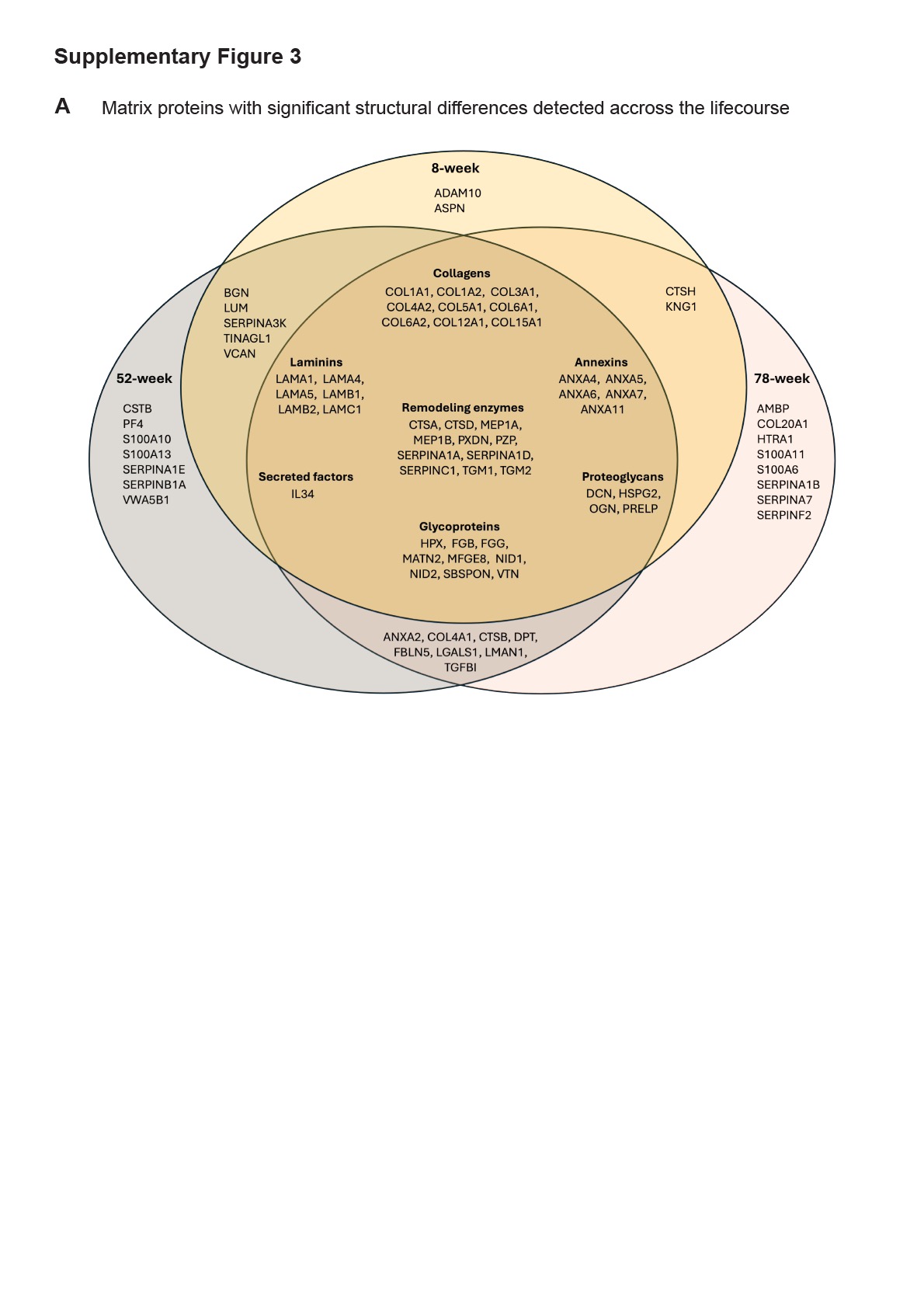
**

**Supplementary Figure 3: A**) Kidney matrix proteins identified by PLF analysis with statistically significantly structure-associated differences between 8-week (middle), 52-week (left) and 78-week (right) compared to 22-week. Central overlap shows a total of 45 matrix proteins were identified as significantly structurally different in 8-, 52-, and 78-week mice relative to 22-week mice.
